# Clinicopathological characteristics of patients with carotid body tumor with cervical lymph node metastasis: A retrospective study of 10 cases and review of the literature

**DOI:** 10.1097/MD.0000000000030379

**Published:** 2022-09-09

**Authors:** Liu Yang, Wen Li, Hongying Zhang, Lingyu Yu, Meijun Zheng

**Affiliations:** a Department of Otolaryngology Head & Neck Surgery, West China Hospital, Sichuan University, Chengdu, Sichuan 610041, People’s Republic of China; b Department of Pathology, West China Hospital, Sichuan University, Chengdu, Sichuan, People’s Republic of China.

**Keywords:** cervical lymphatic metastasis, computer-aided 3D visualization technology, malignant carotid body tumor, pathology, radiography, selective neck dissection

## Abstract

Carotid body tumor (CBT), also known as carotid body chemoreceptor tumor or nonchromaffin paraganglioma, originates from the chemoreceptor behind the common carotid artery bifurcation in the carotid sheath. Most CBTs are benign. Malignant CBT (MCBT) is extremely rare, and cervical lymph node metastasis (CLNM) is usually regarded as a manifestation of malignant behavior. The association between CLNM, pathological features of the primary lesion, clinical manifestations, and prognosis deserves further investigation. The clinical materials of 133 patients with CBT who underwent total resection of the primary tumor and concomitant selective neck dissection (SND) from February 2002 to June 2018 in a single center were reviewed. Postoperative histopathology confirmed CLNM in 10 cases (10/133); clinical manifestations, pathological and imaging characteristics, and treatment outcome data were reviewed and analyzed. The average patient age was 50.5 years, with a female sex tendency (7/10). The mean and median follow-up periods of all cases were 6.9 years and 7 years, respectively. Nine patients (9/10) survived; one patient died of multiple systemic metastases 10 months after surgery when the tumor metastasized to the bilateral breast and other organs in an orderly manner. None of the patients had local recurrence, but postoperative residual lesions were detected by computer-aided 3-dimensional (3D) visualization computerized tomography in one (1/10). Most CBT cases with CLNM displayed adverse features, especially in patients without distant metastases. Immunohistochemically, the patient with distant metastases was negative for S-100, synaptophysin (Syn), and succinate dehydrogenase B (SDHB) expression. Most patients with CBT with CLNM have a good prognosis. Breast metastasis is an exceedingly rare manifestation of MCBT. Despite some association between clinical biological and histological malignancies in CBT with CLNM, the association seems to be vague in cases involving distant metastasis. The combination of certain immunohistochemical indicators (S-100, Syn, and SDHB) might be valuable for predicting the occurrence of distant metastasis. Computer-aided 3D visualization technology might be helpful for the diagnosis and postoperative follow-up of MCBT. Simultaneous SND can remove potentially metastatic lymph nodes and facilitate diagnosis and treatment.

## 1. Introduction

Carotid body tumor (CBT), also known as carotid body chemoreceptor tumor or nonchromaffin paraganglioma, originates from the chemoreceptor behind the common carotid artery bifurcation of the carotid sheath and was first reported by Marchandin in 1891.^[1]^ CBT is commonly regarded as a benign lesion that may go unnoticed due to its nonfunctional nature. Malignant CBT (MCBT) is rare, and there is no consensus on its definition; the incidence of MCBT in CBT based on previous literature varies from 2% to 18%.^[2,3]^ Some scholars have attempted to define malignancies based on histological markers, including central necrosis, vascular infiltration, and mitotic/nuclear atypia.^[4]^ However, it is difficult to judge benign or malignant paragangliomas only by histological morphology because histological behavior is not always consistent with clinical and biological behaviors.^[2]^ An increasing number of investigators believe that malignancy should be considered only when metastasis occurs in sites lacking CBT cells, such as cervical lymph nodes or distant sites (the liver and vertebrae, etc).^[5,6]^ In familial or sporadic cases that were recently characterized, succinate dehydrogenase (SDH) mutations may be responsible for transforming a malignant paraganglioma.^[7]^ We support that the malignant potential of CBT is confirmed by their metastasis rather than their histological features. Nevertheless, the relationship between metastasis and adverse histological features needs further confirmatory research.

CBT with cervical lymph node metastasis (CLNM) is the typical malignant biological behavior between malignant histological morphology and distant metastasis. Paraganglioma metastasizing to regional lymph nodes may exhibit indolent clinical behavior. as suggested by Javidiparsijani et al^[8]^ As lymph node dissection is not indispensable in CBT resection, CBT with CLNM is often diagnosed through cervical lymph node biopsy during the second surgery in cases of recurrence.^[9]^ Nevertheless, there are few studies on the relationship between CLNM and the adverse pathological characteristics, clinical manifestations, and prognosis of primary lesions. We retrospectively analyzed 10 CBT cases with CLNM, which were confirmed pathologically after CBT resection with ipsilateral selective neck dissection (SND). This study reports some adverse histological features of these ten patients and provides clinical manifestation and outcome analyses of these cases. The role of synchronous SND in CBT therapy is also discussed.

## 2. Materials and Methods

The clinical materials of 133 patients with CBT who underwent total resection of the primary mass and SND simultaneously from February 2002 to June 2018 in West China Hospital of Sichuan University were reviewed. The above data were obtained by searching for the 2 Chinese medical terms (CBT and Neck dissection) in the electronic medical records system, excluding the cases with asynchronous neck dissection. After introducing the term (lymph node metastasis) in the above retrieval, it revealed that only 10 cases were identified as CBT with cervical CLNM and showed characteristic histopathologic features. Clinical and radiologic features were collected from electronic medical records, and the gross specimens and intraoperative details were reviewed through intraoperative photographs and videos. Information regarding subsequent treatment and follow-up was obtained from the patients via telephone conversation and outpatient visits.

A professional surgeon evaluated the gross surgical specimens. The hematoxylin and eosin slides of 10 cases were reviewed by a pathologist who assessed adverse pathologic features related to malignant behavior, including incomplete capsule, tumor embolus, nuclear atypia, necrosis, venous invasion, nerve invasion, and the extent of tumor invasion. Moreover, formalin-fixed paraffin-embedded tissue blocks were retrieved for immunohistochemical studies, and the proliferation activity index of Ki-67 in the paraffin sections was assessed.

Descriptive analysis methods were used to analyze those data and pertinent literature was also reviewed. This study was approved by the Institutional Review Board and Ethics Committee of the West China Hospital of Sichuan University.

## 3. Results

### 3.1. Clinical features

The local lymph node metastasis rate of CBTs was approximately 7.5% (10/133). The 10 patients (7 females and 3 males) ranged in age from 27 to 72 years, with a mean of 50.5 years. The disease course ranged from 1 to 18 years, with an average of 4.8 years. None of the 10 individuals had a family history of CBT, and all of them lived in areas lower than 2000 meters above mean sea level. Clinically, all the lesions presented as a nontender and slow-growing mass on the unilateral neck region (6 left-sided and 4 right-sided); two patients experienced one postoperative relapse prior to the index admission. The SND scope was expanded if the tumor exceeded the routine levels (II, III, and IV), which occurred in Case 4 and Case 10 (Table 1).

**Table 1:** Clinical characteristics, treatment and follow-up of patients with malignant carotid body tumors.

Patient	Age (yr)	Sex	DC^1^ (yr)	SC^2^	Presenting feature^3^	Extent of lymph node dissection	Ratio of metastatic lymph nodes	Postoperative complications	Further treatment	Follow-up duration (yr)	Follow-up status
Case 1	61	M	3	III	Unilateral headache	II,III,IV	1/10	None	None	7	Disease-free
Case 2	44	F	4	III	None	II,III,IV	2/10	Postoperative hemorrhage	None	8	Disease-free
Case 3	27	F	1	II	None	II,III,IV	3/8	None	Chemotherapeutic and interventional therapy (four cycles)	10M^5^	Deceased due to distant metastasis
Case 4	65	M	4	III	None	II,III,IV,V	2/10	Hemiglossoplegia; dysphonia and bucking	None	9	Disease-free
Case 5	29	F	4	III	Hacking cough and slight dizziness	II,III,IV	3/18	Hoarseness and bucking	None	7	Disease-free
Case 6	53	M	4	III	Unilateral vocal cord paralysis; recurrent lesion	II,III,IV	6/15	None	Reoperation and gamma knife radiosurgery	10	Alive with residual lesions
Case 7	72	F	2	II	Recurrent lesion	II,III,IV	2/13	None	None	8	Disease-free
Case 8	59	F	5	III	None	II,III,IV	1/9	Hemiglossoplegia	None	7	Disease-free
Case 9	55	F	3	II	None	II,III,IV	1/10	None	None	7	Disease-free
Case 10	40	F	18	III	Sleep apnea	I to VI	1/11	Hemiglossoplegia and hoarseness	None	5	Disease-free

1: disease course; 2: Shamblin classification; 3: other presenting features except a nontender and slow-growing mass; 4: number of metastatic lymph nodes/total number of dissected lymph nodes; 5: 10 months.

**Figure 1.**
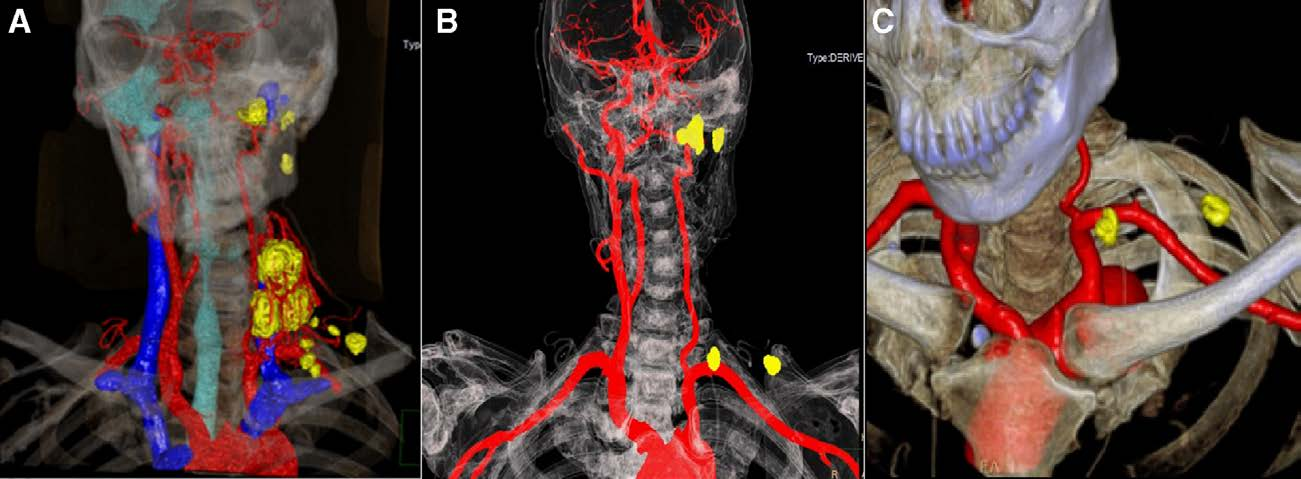
(A) Computer-aided 3D visualization CT of the recurrence and metastases (Case 6), A-P view demonstrating a recurrence (mainly fused metastases) with abundant blood supply main from the subclavian artery and vertebral artery. The yellow scattered nodules were lymph nodes but not all metastatic. (B and C) Postoperative residual lesions were located in skull base and thoracic cavity (Case 6). 3D = 3-dimensional, CT = computerized tomography.

All patients underwent contrast-enhanced magnetic resonance angiography (CE-MRA) or contrast-enhanced computed tomography angiography (CE-CTA). Typical CE-MRA images of CBT were obtained in all cases. In general, typical CE-MRA results revealed a round-like or dumbbell-shaped mass at the carotid bifurcation on T1-weighted images that showed an isointense signal; a hyperintense signal on T2-weighted images and the lesion were distinctly enhanced on an enhanced scan. The “pepper and salt” sign due to the flow void effect was obvious in 8 of the 10 cases. In the other 2 cases, we observed that the cross-sectional CE-MRA image of the tumor had no-signal areas in the center. 3-dimensional (3D) reconstruction of the tumor commonly revealed a “goblet” sign with tortuous and disordered vascular clusters, especially in this series of Shamblin III cases. In one case, there was a separate nodule with a hyperintense signal on T2-weighted imaging, which we supposed to be a scattered tumor nodule that was finally pathologically proven to be a metastatic lymph node. A metastatic lymph node was identified by preoperative CE-CTA for Case 5. Computer-aided 3D visualization computerized tomography was conducted in 2 cases. We found multiple ipsilateral lesions in the neck and at the skull base in Case 6, and the large foci presumed to be recurrence were pathologically proven to be a combination of recurrent tumors and fused metastatic lymph nodes. In addition, not all scattered lymph nodes were confirmed to be metastases (Fig. 1A). Color doppler ultrasonography was typically used to review the patency of blood vessels in patients who underwent intraoperative blood vessel reconstruction (Cases 1, 4, and 5). Interestingly, one suspicious metastatic lymph node detected on preoperative imaging was confirmed to be metastatic thyroid papillary carcinoma using an intraoperative frozen specimen.

### 3.2. Treatment and outcome

All ten patients underwent en bloc resection of CBT and SND simultaneously. Five patients experienced postoperative complications, mainly neurological dysfunction, which were completely or partially restored during follow-up because of nerve reconstruction or nerve grafting application. All patients survived, except for the patient in Case 3, who died of multiple systemic successive metastases involving the bilateral breast, pulmonary, left adnexa uteri, and left adrenal at ten months after surgery. The patient in Case 3 underwent 4 rounds of chemotherapy and one round of interventional therapy after surgery. Case 6 was treated with gamma knife radiosurgery due to residual lesions at the base of the skull after 2 resections (no further progression during the follow-up) (Fig. 1B and C). The mean and median follow-up periods of all cases were 6.9 years and 7 years, respectively (ranging from 10 months to 10 years). All clinical characteristics, treatments and follow-up are documented in Table 1.

### 3.3. Pathologic features

#### 3.3.1. Morphological and histological findings.

**Figure 2.**
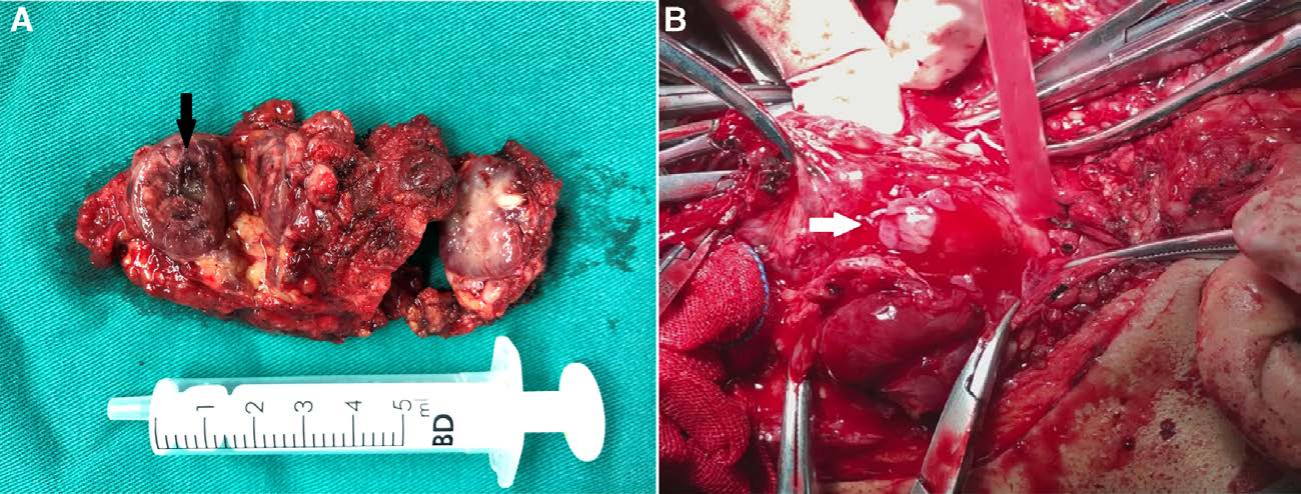
(A) Gross specimen of recurrence metastatic foci with bleeding and cystic changes (black arrow) (Case 6). (B) The tumor thrombus (white arrow) removed from the internal jugular vein (Case 7).

In the gross surgical specimens, the diameters of the tumors ranged from 2.5 to 13 cm, with an average of 5.47 cm. Most specimens comprised an entity with nodular changes (8/10), and the sections were variegated with gray–red, gray, or taupe; bleeding and cystic changes occurred in 2 recurrent cases (Fig. 2A). The Shamblin classification of the tumors are shown in the Table 2. Tumor thrombi in the blood vessels of the main body tumor, lymph nodes or internal jugular vein were found in 5 cases (5/10) (Fig. 2B).

**Table 2:** Morphological and adverse histological features of malignant carotid body tumors.

Patient	Tumor size (cm)	Nodular lesion	Incomplete capsule	Nuclear atypia	Necrosis	Venous invasion	Nerve invasion	Tumor thrombus
Case 1	4 × 4 × 3.5	+	+	+	+	+	−	−
Case 2	3.7 × 3.6 × 4.2	+	+	−	+	+	+	−
Case 3	2.5 × 2.5 × 2	−	−	+	−	+	−	−
Case 4	11 × 9 × 5	+	+	+	+	+	+	+
Case 5	4.5 × 4 × 4	+	+	−	+	+	+	+
Case 6	5 × 3.5 × 3	+	+	+	+	+	+	+
Case 7	3 × 2.5 × 2.5	+	−	+	−	+	−	+
Case 8	5 × 5 × 3	+	+	−	−	+	−	−
Case 9	2.5 × 2.5 × 2	−	+	+	−	+	−	−
Case 10	13 × 10 × 9	+	+	+	+	+	+	+

+: present; −: absent.

**Figure 3.**
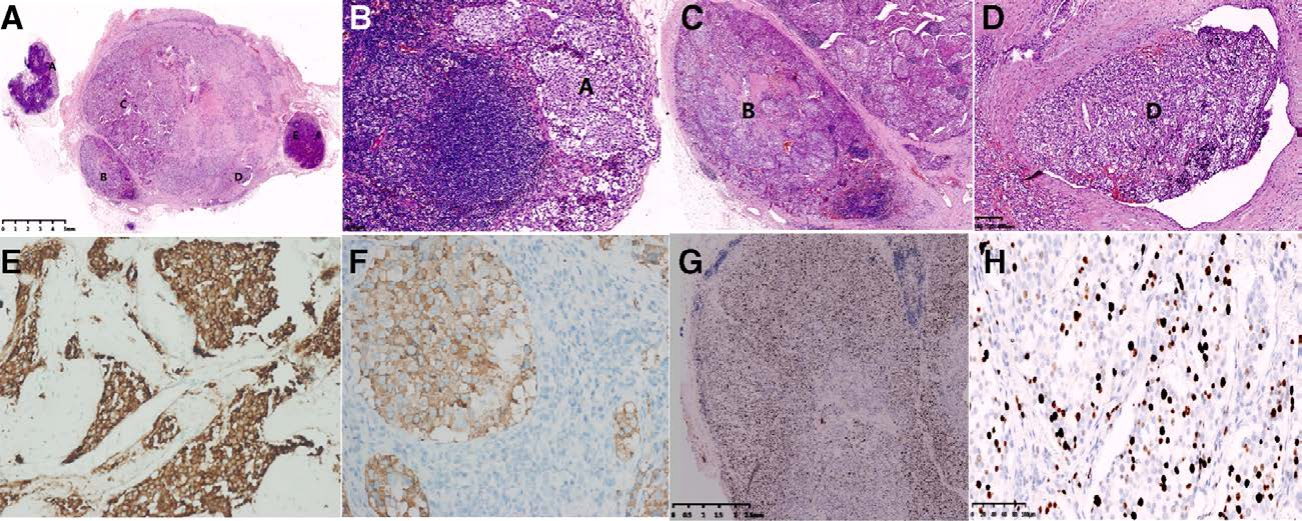
(A–D) (Case 6): fused lymph nodes with metastasis lymph nodes (A, B, E), the largest metastatic lymph node (C), tumor thrombus in the lymph node (D) (E: hematoxylin-eosin, original magnification: ×50). (B) Enlarged view of A-A (hematoxylin-eosin, original magnification: ×100). (C) Enlarged view of A-B showing metastasis with typical zellballen configuration (hematoxylin-eosin, original magnification: ×100). (D) Enlarged view of a-D showing tumor thrombus with typical zellballen configuration in the lymph nodes (hematoxylin-eosin, original magnification: ×100). (E and F) (Case 7), tumor cells showing positivity to Syn and Cga by immunohistological staining respectively; (G and H) (Case 3) showing that the rate of MIb-1 positivity was much higher percentage in the lymphatic foci than in the primary tumor, Mib-1 positivity reached to approximately 30% (immunohistochemistry, original magnification: 5a: ×40,5b: ×200). Cga = chromogranin, Syn = synaptophysin.

Histologically, the tumors were composed of chief cells and sustentacular cells. The chief cells were seen in groups referred to as zellballen, surrounded by extensive vascular sinusoids. Morphologic features showed nerve infiltration in half of the specimens. Incomplete capsule, nuclear atypia, and necrosis were present in 80%, 70%, and 60% of cases, respectively (Table 2). The ratio between metastatic and total lymph nodes was 0.1 to 0.4. Metastatic lymph nodes with fused lymph nodes were common in recurrent cases (Fig. 3A–D). However, the cellular atypia in the lymph node metastasis was not more obvious than that in the main body tumor. In Case 3, metastases in breast tissue were confirmed histologically.

#### 3.3.2. Immunophenotypic findings.

An array of relevant immunohistochemistry markers were analyzed (Table 3). All chief cells were variably positive for markers chromogranin and negative for pancytokeratin and epithelial membrane antigen (Fig. 3E and F). The rate of Ki67 positivity varied and reached a much higher percentage in lymphatic foci in comparison with the primary tumor (Fig. 3G and H).

**Table 3:** Immunohistochemical differences in carotid body tumors with cervical lymph node metastasis.

Patient	S100 (sustentacular cells)	CgA	Syn	PCK	EMA	SDHB	NSE	Ki67
Case 1	+	+	+	−	−	−	+	+, ≤5%
Case 2	+	+	+	−	−	+	+	+, 6%
Case 3	−	+	−	−	−	−	−	+, 30%
Case 4	+	+	+	−	−	+	+	+, 10%
Case 5	+	+	+	−	−	+	−	+, 7%
Case 6	+	+	+	−	−	+	−	+, 20%
Case 7	−	+	+	−	−	−	+	+, ≤5%
Case 8	−	+	+	−	−	+	−	+, ≤5%
Case 9	+	+	+	−	−	+	+	+, ≤5%
Case 10	+	+	+	−	−	+	+	+, 11%

+: positive; −: negative.

CgA = chromogranin; EMA = epithelial membrane antigen; PCK = pancytokeratin; SDHB = succinate dehydrogenase B; Syn = synaptophysin; NSE = neuron-specific enolase.

## 4. Discussion

CBTs are often diagnosed by their location, clinical symptoms, and imaging findings. Preoperative imaging diagnosis is essential for diagnosis and treatment. CE-CTA, CE-MRA, and 3D reconstruction of vessels are commonly used for CBT diagnosis. Computer-aided 3D visualization technology has been widely applied to plastic and reconstructive surgery, hepatic surgery, and robotic surgery, yet there are few reports for CBT. At present, the application of this technique in head and neck diseases is mostly limited to the reconstruction and evaluation of the maxillofacial support framework, such as the reconstruction of the mandible, maxilla, and orbital wall, etc.^[10,11]^ Although there are a handful of reports suggesting the technology in the head and neck can assess the soft tissue and display complicated neural network structures that have advantages, more research was needed.^[12,13]^ However, Chen et al^[14]^ recently reported its application in thyroid surgery, suggesting that this technique has great potential for diagnosing and treating neck soft tissue tumors. Mediouni et al^[15]^ found multifocal lesions to be characteristic of MCBT, while traditional imaging techniques cannot adequately detect and display the multiple lesions as well as distinguish the properties of lymph nodes (hyperplastic or neoplastic). According to our study, the 3D model can reveal the relationship between multiple lesions and large vessels and demonstrate residual lesions, though it does not appear to differentiate between metastatic fused lymph nodes and single isolated lymph node lesions. Therefore, computer-aided 3D visualization technology can intuitively display the spatial distribution of lymph nodes involved or lesions so that it could be instructive for surgeons on the extent of SND and surgical risk assessment. In short, the application of computer-aided 3D visualization technology in CBT has important clinical significance for treatment plans and prognosis assessment.

CBTs are classified as noninvasive, invasive, and metastatic histologically. Whether aggressive histological findings in CBTs constitute malignancy remains a controversial issue. Some researchers have discovered that even in some cases with distant metastasis, malignant histological features might not exist in the original focus.^[5]^ The same phenomenon was observed in our case series. We also observed significant tumor cell atypia in the primary lesion in case 3, although there were few adverse histological features. Most CBTs with CLNM display adverse features both in the gross specimens and microscopic structures. Gu et al^[16]^ found that compared with benign CBTs, MCBTs have a more advanced Shamblin classification and larger tumor size, and this conclusion is consistent with our findings. In our study, the tumor size of gross specimens was larger than 50 cm^3^ in 7 patients (7/10), and 8 cases (8/10) were categorized as Shamblin III. However, the only case (Case 3) with distant metastasis had the smallest volume. In addition, the cross-section of benign CBT specimens was usually neat and smooth, but there was a high proportion of nodular changes (8/10), except in Case 3 and Case 9. This finding seems to suggest that both larger size and nodular cut surface are risk factors for CBT, which preferentially metastasizes to the regional lymph nodes. However, these speculations did not apply to the case of metastasis. Moreover, bleeding and cystic changes in the tumor entity might be associated with recurrent lesions.

Histologically, venous invasion might be an adverse pathologic feature related to lymph node metastasis, as it was present in all cases. Although incomplete capsule and nuclear atypia may occur in CBT, whether benign or malignant, they occurred in more than half of the cases we studied, and thus the 2 manifestations are worthy of notice. It has been reported that necrosis is a characteristic of MCBT.^[4]^ Necrosis was found in 5 cases (5/10), including all cases of recurrence (2/10), in this study. Therefore, relapse is a clinical manifestation of MCBT. Tumor thrombus is one of the complications of malignant tumors. It refers to the aggregation of tumor cells in blood vessels or lymphatic vessels, similar to thrombosis. Tumor thrombi may cause abnormal coagulation function and blood flow disorders, and the presence of a tumor thrombus suggests the possibility of distant metastasis. Tumor thrombi in the head and neck region are rare, with most being caused by squamous cell carcinoma or thyroid carcinoma.^[17]^ Although the formation of tumor thrombi is theoretically possible due to the vascular erosion of CBT, it has rarely been reported. Tumor thrombus formation was detected in 5 patients, including those whose thrombus was removed intraoperatively (Case 6). In our study, the tumor thrombus seemed to be related to the recurrence and size of the CBT, but no distant metastasis was found during follow-up in these cases. In addition, some researchers have suggested that CBT with a sclerosing variant may have a more aggressive histological behavior to cause CLNM.^[8]^ Finally, the case with distant metastases seemed to show a minor association with malignant histopathology compared to regional metastasis; only nuclear atypia and moderate venous invasion were discovered.

Immunohistochemistry is mainly applied to distinguish MCBT from other malignant tumors. The chief cells of CBT are frequently positive for chromogranin and Syn and negative for epithelial membrane antigen, parathyroid hormone, pancytokeratin, thyroid transcription factor, HMB-45, and renal cell carcinoma marker. Hence, CBT can be distinguished from carcinoid carcinoma, medullary thyroid carcinoma, anaplastic carcinoma, metastatic melanoma, and renal cell carcinoma. In most CBTs, sustentacular cells are positive for S-100 protein, but some researchers have found that MCBTs showed less reactivity than benign CBTs.^[18,19]^ Studies have also shown that CBTs are more likely to be malignant if on average fewer than 5 neuropeptides are expressed.^[20]^ Neuron-specific enolase is an isoform of enolase, a glycolytic enzyme found in neuroendocrine cells and tumors, and it is positive in most CBTs. Although 60% of our cases were positive, Kumaki et al^[18]^ reported that expression of neuroendocrine markers does not appear to correlate with malignant potential malignant cases. It has been reported that paraganglioma from a patient with an SDH germline mutation showed loss of succinate dehydrogenase B (SDHB) expression, and SDH mutations may be related to malignant paraganglioma transformation.^[21]^ Therefore, we speculate that Cases 1, 3, and 7 might involve SDH mutation. Although the positive rates of the 3 indicators (S-100, Syn, and SDHB) in our case series showed slight differences from those in previous benign cases, the results need further statistical analysis. Interestingly, the case with distant metastases was negative for all 3, while the results did not exist in other cases. We speculate that the combination of these 3 antibodies may help predict the diagnosis of distant metastases. The rate of Ki67 positivity may not be a good indicator for the malignant grade of MCBT; in our study, it showed a wide range expression rate from 5% to 30% in all metastatic cases. For instance, Ki-67 was much higher in the metastatic lesion than in the primary lesion in Case 6, but it was expressed at almost the same level between the primary lesion and metastatic foci in the other cases. Kumaki et al^[18]^ suggested that Ki-67 immunostaining is a valuable adjunct marker to predict malignant behavior in these tumors. Regardless, less malignant biological behavior should not be expected with a metastatic carotid body tumor with high Ki-67 expression.

Although some scholars believe radiotherapy should be considered the first-line treatment of choice for head and neck paragangliomas,^[22]^ surgery is still the mainstay therapeutic option for CBT and MCBT because MCBT diagnosis is often established after surgery. Lymph node resection, whether it is malignancy diagnosis oriented or just needed for exposure of the tumor, is essential. A surgeon is not able to reach the dissection plane between the CBT and the carotid artery without resection of the lymph nodes, especially those around the common facial vein (level II). Intraoperative lymph nodes frozen for CBT surgery are not routinely carried out even in a teaching hospital or scientific institution, as the morbidity is extremely low. Radiotherapy can achieve local lesion control rather than eradicating lesions.^[23]^ Local regional lymph node dissection remains controversial in the literature. Some suggest that concomitant lymph node dissection does not appear to add value in the absence of clinical suspicion for malignancy, yet a recent study showed that intraoperative dissection of level IIA lymph nodes in CBT patients might contribute to the diagnosis and prognosis of MCBTs.^[24,25]^ Distant spread was rare in our reported cases. When the primary tumor and locoregional lymph nodes were excised, neither further regional lymph node metastasis nor distant spread was observed during long-term follow-up in most cases. In our opinion, despite no preoperative radiographic evidence of CLNM, SND should be conducted conventionally to expose the CBT main body and evaluate the prognosis. Concerning the scope of SND, we recommend II, III, and IV levels as common areas rather than the IIA level because when the CBT is large enough, IIA level lymph node resection is obviously not sufficient. In general, CBT is removed in an en bloc manner; thus, any solitary nodules outside the main-body tumor should be separately collected for pathological examination, as lymph node metastasis was found deep to the carotid artery once in this case series. Overall, SND is likely to be a satisfactory treatment modality for local regional control, though it is still not radical for good and all for local control. It cannot prevent distant metastases as well. In comparison with unplanned reoperation or postoperative adjuvant radiotherapy soon after CBT surgery, concomitant selected lymph node dissection is rational for doctors and patients, both with regard to reducing injury and cost. For patients with multiple systemic metastases or unresectable lesions, comprehensive treatment is recommended.^[26]^ However, this study is a retrospective study based on the medical record series, which only provides some possible directions for the study of this rare disease, so we look forward to further prospective studies to verify these characteristics.

## 5. Conclusions

In conclusion, most patients with CBT with CLNM have a good prognosis. Computer-aided 3D visualization technology might be a new tool for the diagnosis and follow-up of MCBT. Breast metastasis is an exceedingly rare manifestation of MCBT. We suppose that some degree of association exists between malignant biological behavior and adverse histological manifestations in CBT with CLNM, but this association seems to be vague in cases with distant metastasis. The combination of some immunohistochemical indicators (S-100, Syn, and SDHB) might be significant for predicting the occurrence of distant metastasis. Moreover, simultaneous SND can help to remove potential metastatic lymph nodes and facilitate further diagnosis and treatment.

## Acknowledgments

This manuscript has been edited by American Journal Experts.

## Author contributions

**Conceptualization:** Wen Li.

**Data curation:** Liu Yang, Lingyu Yu, Meijun Zheng.

**Investigation:** Liu Yang.

**Methodology:** Liu Yang.

**Project administration:** Liu Yang.

**Resources:** Wen Li, Hongying Zhang.

**Supervision:** Wen Li, Hongying Zhang.

**Visualization:** Lingyu Yu.

**Writing – original draft:** Liu Yang.

**Writing – review & editing:** Wen Li.
